# [1-(Carb­oxy­meth­yl)cyclo­hex­yl]methanaminium nitrate

**DOI:** 10.1107/S1600536811001267

**Published:** 2011-01-29

**Authors:** Elise J. C. de Vries, Caryn Gamble, Ahmed Shaikjee

**Affiliations:** a Molecular Science Institute, School of Chemistry, University of the Witwatersrand, PO WITS, 2050 Johannesburg, South Africa

## Abstract

The title compound, C_9_H_18_NO_2_^+^·NO_3_^−^, is an anhydrous nitrate salt of gabapentin, which is formed serendipitously in the presence of selected non-coordinating metals. The crystal structure involves extensive hydrogen bonding between the –NH_3_^+^ and –COOH groups and the nitrate anion.

## Related literature

For related structures, see: Ibers (2001 ▶); Ananda *et al.* (2003 ▶); Reece & Levendis (2008 ▶); Braga *et al.* (2008 ▶); Fabbiani *et al.* (2010 ▶). For the role of γ-amino­butyric acid (GABA) as an inhibitory neurotransmitter, see: Bowery (1993 ▶). Gabapentin is used as a neuroleptic drug in the treatment of epilepsy (Taylor, 1993 ▶) but its applications have been extended to the treatment of neuropathic pain (Magnus, 1999 ▶).
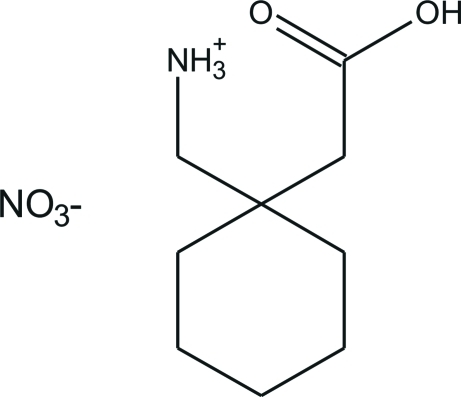


## Experimental

### 

#### Crystal data

C_9_H_18_NO_2_^+^·NO_3_^−^
*M* *_r_* = 234.25
Orthorhombic, 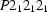

*a* = 8.1743 (8) Å
*b* = 11.5945 (11) Å
*c* = 12.0396 (9) Å
*V* = 1141.08 (18) Å^3^
*Z* = 4
Mo *K*α radiation
μ = 0.11 mm^−1^
*T* = 173 K
0.65 × 0.15 × 0.14 mm

#### Data collection

Bruker APEXII CCD diffractometer
5654 measured reflections
1448 independent reflections
1278 reflections with *I* > 2σ(*I*)
*R*_int_ = 0.044

#### Refinement

*R*[*F* ^2^ > 2σ(*F* ^2^)] = 0.033
*wR*(*F* ^2^) = 0.084
*S* = 1.06
1448 reflections
146 parameters
H-atom parameters constrained
Δρ_max_ = 0.22 e Å^−3^
Δρ_min_ = −0.14 e Å^−3^

### 

Data collection: *APEX2* (Bruker, 2005 ▶); cell refinement: *SAINT-NT* (Bruker, 2005 ▶); data reduction: *SAINT-NT*; program(s) used to solve structure: *SHELXS97* (Sheldrick, 2008) ▶; program(s) used to refine structure: *SHELXL97* (Sheldrick, 2008) ▶; molecular graphics: *X-SEED* (Barbour, 2001 ▶; Atwood & Barbour, 2003 ▶); software used to prepare material for publication: *X-SEED*.

## Supplementary Material

Crystal structure: contains datablocks global, I. DOI: 10.1107/S1600536811001267/pb2049sup1.cif

Structure factors: contains datablocks I. DOI: 10.1107/S1600536811001267/pb2049Isup2.hkl

Additional supplementary materials:  crystallographic information; 3D view; checkCIF report

## Figures and Tables

**Table 1:** Hydrogen-bond geometry (Å, °)

*D*—H⋯*A*	*D*—H	H⋯*A*	*D*⋯*A*	*D*—H⋯*A*
N1—H1*A*⋯O5^i^	0.91	2.02	2.828 (2)	147
N1—H1*A*⋯O4^i^	0.91	2.41	3.215 (2)	147
N1—H1*A*⋯N2^i^	0.91	2.58	3.465 (3)	164
N1—H1*B*⋯O4^ii^	0.91	2.06	2.951 (3)	168
N1—H1*B*⋯O3^ii^	0.91	2.47	3.022 (2)	119
N1—H1*B*⋯N2^ii^	0.91	2.60	3.390 (3)	146
N1—H1*C*⋯O1	0.91	1.90	2.760 (2)	157
O2—H2*C*⋯O5	0.84	1.81	2.646 (2)	175
O2—H2*C*⋯N2	0.84	2.60	3.376 (2)	154

Symmetry codes: (i) 
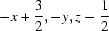
; (ii) 
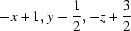
.

**Fig. 1.**
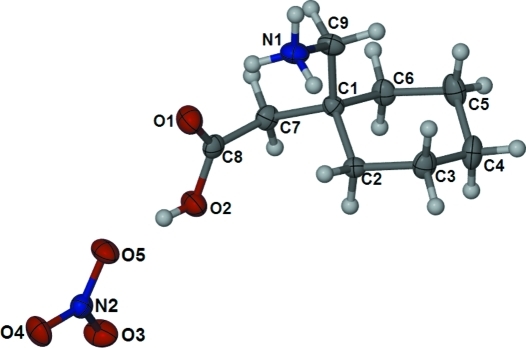
The atomic numbering scheme of complex (I). Displacement ellipsoids are drawn at 50% probability level.

**Fig. 2.**
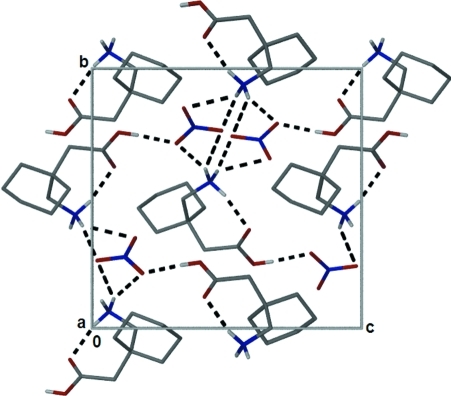
Projection of the unit cell of complex (I) down the a axis. All hydrogen atoms removed, except those involved in hydrogen bonding. Hydrogen bonds are indicated as dashed lines.

## supplementary crystallographic information

### Comment

The role of γ-aminobutyric acid (GABA) as an inhibitory neurotransmitter (Bowery, 1993) has stimulated research on the synthesis of GABA analogues as potential central nervous system agents. One of these analogues is the amino acid gabapentin [1-(aminomethyl)cyclohexaneacetic acid] which is commercially available as Neurotin. Gabapentin is used as a neuroleptic drug in the treatment of epilepsy (Taylor, 1993) but its applications have been extended to the treatment of neuropathic pain (Magnus, 1999). Gabapentin is widely studied and already four polymorphic forms of the drug are known, three polymorphic forms under ambient conditions (Ibers, 2001; Reece and Levendis, 2008; Braga *et al.*, 2008) and a fourth at high pressure (Fabbiani *et al.*, 2010). The present paper reports on the formation of an anhydrous gabapentin nitrate salt, complex (I).

Complex (I) was obtained serendipitously when investigating the possibility of producing novel metal complexes with gabapentin. Lithium-, chromium-, indium-, iron- and aluminium nitrate were used in an attempt to make metal complexes. However analysis of the crystalline materials revealed a gabapentin nitrate salt was obtained in each case. The atomic numbering scheme of the gabapentin nitrate complex, C_9_H_18_NO_2_^+^.NO_3_^-^, is shown in Fig. 1. Complex (I) crystallizes in the orthorhombic space group *P*2_1_2_1_2_1_ with a protonated amine group. The cyclohexane ring is in the chair conformation with the ammonium group in the equatorial position. The conformation of the gabapentin molecule is defined by the formation of an intramolecular hydrogen bond between the carboxylate oxygen and one of the hydrogen atoms belonging to the ammonium group (N1—H1C···O1). The crystal packing shows how each nitrate anion links to three adjacent molecules by means of one O—H···O, four N—H···O, two N—H···N and one O—H···N hydrogen-bonding interactions. The donors are the H atoms of the carboxylic acid and amine group, while the acceptors include all three O atoms of the nitrate anion (Fig. 2). Additionally, one nitrate O atom is involved in a weak hydrogen bonding interaction with a symmetry related carbon atom, with a C9—H9B···O3 distance of 3.317 (3) Å and angle of 149°.

### Experimental

Gabapentin was purchased from Sigma-Aldrich. The gabapentin nitrate salt is formed serendipitously by combining gabapentin with one of the following metal salts in 1:1 stoichiometric ratios; lithium-, chromium-, indium-, iron- and aluminium nitrate. The metal salt and gabapentin were dissolved in 0.1 molar nitric acid and allowed to undergo slow evaporation at ambient temperature. It was noted that this complex did not form if the metal salt was removed from the reaction.

### Refinement

All H atoms were positioned geometrically and allowed to ride on their respective parent atoms, with C—H bond lengths of 0.99 (aromatic CH) 1.00 (methine CH), 0.99 (methylene CH_2_) and 0.98 Å (methyl CH_3_), and with *U*_iso_(H) = 1.2 or 1.5 times *U*_eq_(C). In the absence of significant anomalous scattering, Friedel equivalents were merged before the final refinement.

### Crystal data

**Table:**

C_9_H_18_NO_2_^+^·NO_3_^−^	*F*(000) = 504
*M**_r_* = 234.25	*D*_x_ = 1.364 Mg m^−^^3^
Orthorhombic, *P*2_1_2_1_2_1_	Mo *K*α radiation, λ = 0.71073 Å
Hall symbol: P 2ac 2ab	Cell parameters from 1858 reflections
*a* = 8.1743 (8) Å	θ = 3.0–25.8°
*b* = 11.5945 (11) Å	µ = 0.11 mm^−^^1^
*c* = 12.0396 (9) Å	*T* = 173 K
*V* = 1141.08 (18) Å^3^	Plate, colourless
*Z* = 4	0.65 × 0.15 × 0.14 mm

### Data collection

**Table:**

Bruker APEXII CCD diffractometer	1278 reflections with *I* > 2σ(*I*)
Radiation source: fine-focus sealed tube	*R*_int_ = 0.044
graphite	θ_max_ = 27.0°, θ_min_ = 2.4°
φ and ω scans	*h* = −10→8
5654 measured reflections	*k* = −12→14
1448 independent reflections	*l* = −13→15

### Refinement

**Table:**

Refinement on *F*^2^	Primary atom site location: structure-invariant direct methods
Least-squares matrix: full	Secondary atom site location: difference Fourier map
*R*[*F*^2^ > 2σ(*F*^2^)] = 0.033	Hydrogen site location: inferred from neighbouring sites
*wR*(*F*^2^) = 0.084	H-atom parameters constrained
*S* = 1.06	*w* = 1/[σ^2^(*F*_o_^2^) + (0.0451*P*)^2^ + 0.0272*P*] where *P* = (*F*_o_^2^ + 2*F*_c_^2^)/3
1448 reflections	(Δ/σ)_max_ < 0.001
146 parameters	Δρ_max_ = 0.22 e Å^−^^3^
0 restraints	Δρ_min_ = −0.14 e Å^−^^3^

### Special details

**Table:**

Geometry. All e.s.d.'s (except the e.s.d. in the dihedral angle between two l.s. planes) are estimated using the full covariance matrix. The cell e.s.d.'s are taken into account individually in the estimation of e.s.d.'s in distances, angles and torsion angles; correlations between e.s.d.'s in cell parameters are only used when they are defined by crystal symmetry. An approximate (isotropic) treatment of cell e.s.d.'s is used for estimating e.s.d.'s involving l.s. planes.
Refinement. Refinement of *F*^2^ against ALL reflections. The weighted *R*-factor *wR* and goodness of fit *S* are based on *F*^2^, conventional *R*-factors *R* are based on *F*, with *F* set to zero for negative *F*^2^. The threshold expression of *F*^2^ > σ(*F*^2^) is used only for calculating *R*-factors(gt) *etc*. and is not relevant to the choice of reflections for refinement. *R*-factors based on *F*^2^ are statistically about twice as large as those based on *F*, and *R*- factors based on ALL data will be even larger.

### Fractional atomic coordinates and isotropic or equivalent isotropic displacement parameters (Å^2^)

**Table:**

	*x*	*y*	*z*	*U*_iso_*/*U*_eq_	
C1	0.5335 (2)	0.08869 (17)	0.35572 (15)	0.0209 (4)	
C2	0.3870 (2)	0.03596 (17)	0.41650 (16)	0.0221 (4)	
H2A	0.4250	0.0031	0.4879	0.026*	
H2B	0.3076	0.0979	0.4337	0.026*	
C3	0.3003 (3)	−0.0580 (2)	0.35067 (17)	0.0301 (5)	
H3B	0.2046	−0.0860	0.3932	0.036*	
H3A	0.3756	−0.1238	0.3393	0.036*	
C4	0.2437 (3)	−0.0124 (2)	0.23826 (18)	0.0369 (6)	
H4B	0.1597	0.0480	0.2497	0.044*	
H4A	0.1931	−0.0759	0.1953	0.044*	
C5	0.3869 (3)	0.0380 (2)	0.17244 (17)	0.0354 (6)	
H5A	0.4643	−0.0246	0.1530	0.042*	
H5B	0.3456	0.0721	0.1025	0.042*	
C6	0.4764 (3)	0.13032 (18)	0.23945 (16)	0.0272 (5)	
H6B	0.4031	0.1975	0.2490	0.033*	
H6A	0.5732	0.1564	0.1968	0.033*	
C7	0.5943 (3)	0.19806 (17)	0.41759 (16)	0.0255 (5)	
H7A	0.6985	0.2230	0.3833	0.031*	
H7B	0.5136	0.2605	0.4055	0.031*	
C8	0.6213 (3)	0.18538 (18)	0.54059 (17)	0.0243 (5)	
C9	0.6729 (3)	0.0019 (2)	0.33743 (16)	0.0273 (5)	
H9A	0.6386	−0.0538	0.2797	0.033*	
H9B	0.7694	0.0440	0.3086	0.033*	
N1	0.7225 (2)	−0.06315 (16)	0.43807 (14)	0.0290 (4)	
H1A	0.8126	−0.1063	0.4226	0.043*	
H1B	0.6393	−0.1103	0.4596	0.043*	
H1C	0.7460	−0.0128	0.4938	0.043*	
O1	0.7213 (2)	0.12161 (14)	0.58267 (12)	0.0343 (4)	
O2	0.5264 (2)	0.25326 (14)	0.59950 (11)	0.0325 (4)	
H2C	0.5456	0.2434	0.6674	0.039*	
N2	0.4911 (2)	0.27445 (15)	0.87814 (13)	0.0245 (4)	
O3	0.3960 (2)	0.34667 (14)	0.84059 (13)	0.0397 (4)	
O4	0.5086 (2)	0.26085 (14)	0.97989 (11)	0.0351 (4)	
O5	0.5740 (2)	0.21168 (14)	0.81312 (11)	0.0333 (4)	

### Atomic displacement parameters (Å^2^)

**Table:**

	*U*^11^	*U*^22^	*U*^33^	*U*^12^	*U*^13^	*U*^23^
C1	0.0231 (11)	0.0206 (10)	0.0191 (9)	−0.0003 (8)	0.0014 (8)	0.0006 (7)
C2	0.0206 (10)	0.0232 (10)	0.0224 (9)	0.0005 (9)	0.0021 (8)	0.0002 (8)
C3	0.0306 (12)	0.0289 (12)	0.0307 (11)	−0.0083 (10)	−0.0026 (10)	0.0029 (9)
C4	0.0397 (14)	0.0353 (13)	0.0358 (13)	−0.0083 (12)	−0.0138 (10)	−0.0028 (10)
C5	0.0492 (15)	0.0355 (13)	0.0215 (10)	−0.0054 (12)	−0.0070 (10)	0.0010 (9)
C6	0.0330 (13)	0.0274 (11)	0.0212 (10)	−0.0035 (10)	−0.0009 (9)	0.0051 (8)
C7	0.0265 (11)	0.0211 (10)	0.0288 (11)	−0.0043 (9)	−0.0007 (9)	0.0025 (8)
C8	0.0220 (11)	0.0213 (10)	0.0297 (11)	−0.0052 (9)	−0.0004 (9)	−0.0016 (8)
C9	0.0294 (11)	0.0283 (12)	0.0241 (10)	0.0030 (10)	0.0062 (9)	0.0031 (9)
N1	0.0279 (10)	0.0273 (10)	0.0318 (10)	0.0078 (8)	0.0056 (8)	0.0008 (8)
O1	0.0364 (10)	0.0356 (9)	0.0307 (8)	0.0084 (8)	−0.0063 (7)	−0.0017 (7)
O2	0.0340 (9)	0.0381 (9)	0.0254 (7)	0.0089 (8)	−0.0028 (6)	−0.0040 (6)
N2	0.0241 (10)	0.0224 (9)	0.0268 (9)	−0.0001 (8)	−0.0017 (7)	0.0001 (7)
O3	0.0399 (10)	0.0424 (10)	0.0369 (9)	0.0201 (9)	−0.0004 (8)	0.0076 (7)
O4	0.0458 (10)	0.0381 (9)	0.0214 (7)	0.0065 (8)	−0.0056 (7)	−0.0010 (7)
O5	0.0353 (9)	0.0357 (9)	0.0288 (8)	0.0143 (8)	−0.0002 (7)	−0.0053 (7)

### Geometric parameters (Å, °)

**Table:**

C1—C2	1.531 (3)	C6—H6A	0.9900
C1—C9	1.536 (3)	C7—C8	1.504 (3)
C1—C7	1.552 (3)	C7—H7A	0.9900
C1—C6	1.552 (3)	C7—H7B	0.9900
C2—C3	1.522 (3)	C8—O1	1.214 (3)
C2—H2A	0.9900	C8—O2	1.313 (3)
C2—H2B	0.9900	C9—N1	1.484 (3)
C3—C4	1.525 (3)	C9—H9A	0.9900
C3—H3B	0.9900	C9—H9B	0.9900
C3—H3A	0.9900	N1—H1A	0.9100
C4—C5	1.530 (3)	N1—H1B	0.9100
C4—H4B	0.9900	N1—H1C	0.9100
C4—H4A	0.9900	O2—H2C	0.8400
C5—C6	1.528 (3)	N2—O3	1.229 (2)
C5—H5A	0.9900	N2—O4	1.243 (2)
C5—H5B	0.9900	N2—O5	1.266 (2)
C6—H6B	0.9900		
			
C2—C1—C9	112.79 (16)	C5—C6—H6B	108.8
C2—C1—C7	110.32 (16)	C1—C6—H6B	108.8
C9—C1—C7	111.50 (17)	C5—C6—H6A	108.8
C2—C1—C6	108.67 (17)	C1—C6—H6A	108.8
C9—C1—C6	107.29 (16)	H6B—C6—H6A	107.7
C7—C1—C6	105.95 (16)	C8—C7—C1	116.07 (16)
C3—C2—C1	113.65 (16)	C8—C7—H7A	108.3
C3—C2—H2A	108.8	C1—C7—H7A	108.3
C1—C2—H2A	108.8	C8—C7—H7B	108.3
C3—C2—H2B	108.8	C1—C7—H7B	108.3
C1—C2—H2B	108.8	H7A—C7—H7B	107.4
H2A—C2—H2B	107.7	O1—C8—O2	122.55 (19)
C2—C3—C4	110.82 (19)	O1—C8—C7	124.7 (2)
C2—C3—H3B	109.5	O2—C8—C7	112.73 (19)
C4—C3—H3B	109.5	N1—C9—C1	114.76 (16)
C2—C3—H3A	109.5	N1—C9—H9A	108.6
C4—C3—H3A	109.5	C1—C9—H9A	108.6
H3B—C3—H3A	108.1	N1—C9—H9B	108.6
C3—C4—C5	111.1 (2)	C1—C9—H9B	108.6
C3—C4—H4B	109.4	H9A—C9—H9B	107.6
C5—C4—H4B	109.4	C9—N1—H1A	109.5
C3—C4—H4A	109.4	C9—N1—H1B	109.5
C5—C4—H4A	109.4	H1A—N1—H1B	109.5
H4B—C4—H4A	108.0	C9—N1—H1C	109.5
C6—C5—C4	111.14 (18)	H1A—N1—H1C	109.5
C6—C5—H5A	109.4	H1B—N1—H1C	109.5
C4—C5—H5A	109.4	C8—O2—H2C	109.5
C6—C5—H5B	109.4	O3—N2—O4	121.46 (18)
C4—C5—H5B	109.4	O3—N2—O5	120.20 (16)
H5A—C5—H5B	108.0	O4—N2—O5	118.34 (18)
C5—C6—C1	113.72 (17)		
			
C9—C1—C2—C3	−65.3 (2)	C7—C1—C6—C5	−170.79 (19)
C7—C1—C2—C3	169.32 (17)	C2—C1—C7—C8	49.5 (2)
C6—C1—C2—C3	53.6 (2)	C9—C1—C7—C8	−76.7 (2)
C1—C2—C3—C4	−56.9 (3)	C6—C1—C7—C8	166.91 (19)
C2—C3—C4—C5	56.0 (3)	C1—C7—C8—O1	63.3 (3)
C3—C4—C5—C6	−54.9 (3)	C1—C7—C8—O2	−118.1 (2)
C4—C5—C6—C1	54.1 (3)	C2—C1—C9—N1	−49.7 (2)
C2—C1—C6—C5	−52.2 (2)	C7—C1—C9—N1	75.1 (2)
C9—C1—C6—C5	70.0 (2)	C6—C1—C9—N1	−169.31 (18)

### Hydrogen-bond geometry (Å, °)

**Table:**

*D*—H···*A*	*D*—H	H···*A*	*D*···*A*	*D*—H···*A*
N1—H1A···O5^i^	0.91	2.02	2.828 (2)	147
N1—H1A···O4^i^	0.91	2.41	3.215 (2)	147
N1—H1A···N2^i^	0.91	2.58	3.465 (3)	164
N1—H1B···O4^ii^	0.91	2.06	2.951 (3)	168
N1—H1B···O3^ii^	0.91	2.47	3.022 (2)	119
N1—H1B···N2^ii^	0.91	2.60	3.390 (3)	146
N1—H1C···O1	0.91	1.90	2.760 (2)	157
O2—H2C···O5	0.84	1.81	2.646 (2)	175
O2—H2C···N2	0.84	2.60	3.376 (2)	154

Symmetry codes: (i) −*x*+3/2, −*y*, *z*−1/2; (ii) −*x*+1, *y*−1/2, −*z*+3/2.
